# A negative association between total cholesterol and bone mineral density in US adult women

**DOI:** 10.3389/fnut.2022.937352

**Published:** 2022-09-30

**Authors:** Weihua Fang, Peng Peng, Fangjun Xiao, Wei He, Qiushi Wei, Mincong He

**Affiliations:** ^1^The Third Clinical Medical School, Guangzhou University of Chinese Medicine, Guangzhou, China; ^2^The First Clinical Medical School, Guangzhou University of Chinese Medicine, Guangzhou, China; ^3^Guangdong Research Institute for Orthopedics and Traumatology of Chinese Medicine, Guangzhou, China; ^4^Department of Orthopaedics, The Third Affiliated Hospital, Guangzhou University of Chinese Medicine, Guangzhou, China

**Keywords:** total cholesterol, bone mineral density, osteoporosis, NHANES, cross-sectional study

## Abstract

**Background:**

The association between serum total cholesterol (TC) and bone mineral density (BMD) is still controversial. We aimed to evaluate the association of serum TC with BMD in general US adult women.

**Methods:**

A cross-sectional study consisting of 7,092 (age range 20–85) participants from the National Health and Nutrition Examination Survey (NHANES) database was conducted. Weighted multivariate linear regression analyses were performed to evaluate association between serum TC and lumbar spine BMD. In addition, subgroup and interaction analysis were used in this study.

**Results:**

The serum TC was negatively correlated with lumbar spine BMD after adjusting for confounders. Subgroup analysis found that the strongest negative association mainly exists in women aged over 45 years with body mass index (BMI) < 24.9 kg/m^2^, and this association is not significant in other groups.

**Conclusions:**

This study found that serum TC exhibit an inverse association with lumbar spine BMD in Us women aged over 45 years. The measurement of serum TC may provide information for predicting poor bone health outcomes in these women.

## Introduction

Osteoporosis is a high incidence disease characterized by decreased bone mass, microarchitectural degeneration, and fragility fractures (1). Osteoporosis causes ~1.5 million fractures each year in the United States, the great majority of which occur in postmenopausal women (2). In 1997, the medical expenditures for osteoporotic fractures in the United States were $14 billion, and the cost is expected to reach nearly $ 50 billion by 2040 (3). Bone mineral density (BMD) is an effective indicator for assessing osteoporosis, and low BMD is associated with high fracture risk (4). Therefore, recognizing the risk factors for low BMD is critical for the predicting and prevention of osteoporosis.

There is growing evidence of a strong relationship between BMD and cardiovascular diseases and the metabolic syndrome, with the serum lipid profile possibly playing a key role in this interaction (5–9). As a result, a growing number of studies have examined the relationship between BMD and serum lipids. In a cross-sectional study, Sun C et al. reported that serum total cholesterol (TC) had a large negative impact on BMD in US population (10). Another study from Denmark observed that TC showed significant negative correlation with BMD at the lumbar spine and distal forearm, but not at the hip (11). Also, Trimpou et al. reported that serum TC is an independent risk factor for osteoporotic fracture, and high TC level is a long-term cause of osteoporotic fractures (12). However, a cross-sectional analysis in 136 Caucasian found that higher level of TC was positively associated with BMD of various skeletal sites (13). In addition, several studies have detected no association of serum TC levels with BMD (14–17).

The results of these studies show that the association between serum TC and BMD is still uncertain. Therefore, it's worthwhile to investigate the association between serum TC and BMD to determine whether serum TC can be used to predict the likelihood of osteoporosis or osteopenia. This study aimed to investigate the association between serum TC and lumbar spine BMD in US adult women using data from the National Health and Nutrition Examination Survey (NHANES) database.

## Materials and methods

### Study population

**Figure 1:**
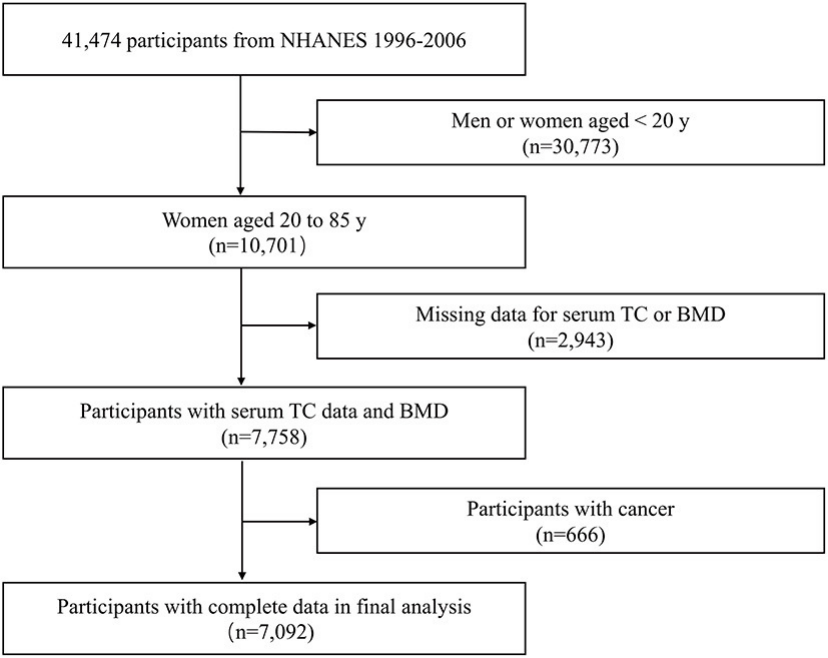
Flowchart of the participants selection.

The NHANES database is a population-based national survey that provided information on the nutrition and health of the American population. The NHANES database is available publicly at www.cdc.gov/nchs/nhanes. Data from 1999 to 2006 in NHANES were combined in our study. Of the 41,474 participants, there were 10,701 women aged 20–85 years, 2,943 had missing data on TC or BMD, and 666 had a cancer diagnosis. After applying these exclusion criteria, 7,092 participants were included in the final analysis (Figure 1).

### Study variables

The independent variable in this study was serum TC. The dependent variable was lumbar spine BMD measured by the dual-energy x-ray (DEXA) scans. The following variables were included in final analysis as covariates: age, race, physical activity, education, body mass index (BMI), triglycerides, HDL cholesterol, serum phosphorus, serum calcium, serum potassium, alkaline phosphatase, LDL cholesterol, phosphorus intake, and protein intake. The examination parts related to clinical and laboratory evaluations were all carried out by well-trained medical experts. The detailed acquisition process and measuring method of each variable are available at www.cdc.gov/nchs/nhanes.

### Statistical analysis

All analyses used weights from the NHANES examination sample adjusted for non-response, non-coverage, and unequal probabilities of selection. In the descriptive analysis, continuous variables were reported as mean ± standard deviation; categorical variables were reported as percentages. Weighted multivariate linear regression models were performed to evaluate the association between serum TC and lumbar spine BMD. Interaction and stratified analyses by age and BMI were performed. *P* < 0.05 (two-sided) was considered statistically significant. Modeling was performed with the EmpowerStats (http://www.empowerstats.com, X&Y Solutions, Inc., Boston, MA) and statistical software packages R (http://www.R-project.org, The R Foundation).

## Results

### Baseline characteristics of participants

**Figure 2:**
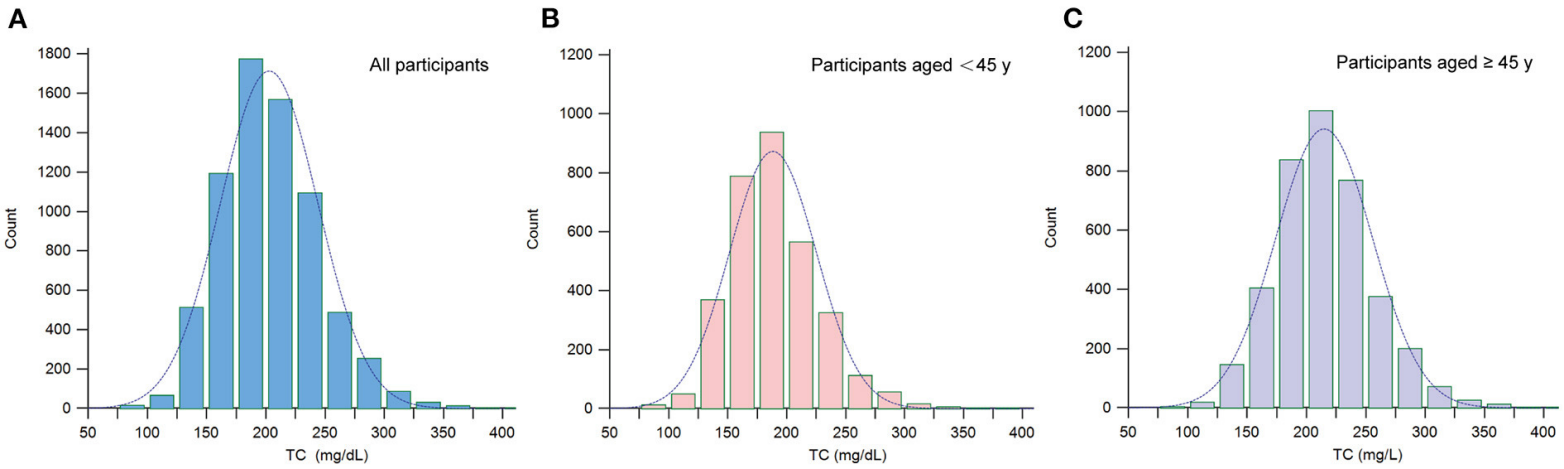
Distribution histogram of serum TC. **(A)** Among all participants; **(B)** Among participants aged < 45; **(C)** Among participants aged ≥45. TC, total cholesterol.

A total of 7,092 women aged 20–85 years were included in this study. The weighted population characteristics of participants by serum TC quartiles (Q1: 82–174 mg/dL; Q2: 175–199 mg/dL; Q3: 200–228 mg/dL; Q4: 229–419 mg/dL) were shown in Table 1. Among different groups of serum TC (quartiles, Q1–Q4), age, race, physical activity, education, BMI, triglycerides, HDL cholesterol, serum phosphorus, serum calcium, serum potassium, alkaline phosphatase, LDL cholesterol, phosphorus intake, protein intake, and lumbar spine BMD are all significantly different. Participants in the top quartile of serum TC were more likely to be older, whites, have lower values of lumbar spine BMD and higher triglycerides, LDL cholesterol, and alkaline phosphatase. In addition, the distribution of serum TC is presented in Figure 2.

**Table 1:** Weighted characteristics of 7,092 participants included in this study.

**Total cholesterol (mg/dL)**	**Q1 (82–174)**	**Q2 (175–199)**	**Q3 (200–228)**	**Q4 (229–419)**	***P* value**
*N*, unweighted	1786	1772	1794	1740	
Age (years)					< 0.0001
Age < 45	30.11 ± 7.30	32.51 ± 7.30	33.75 ± 6.92	35.12 ± 6.21	
Age ≧ 45	57.62 ± 11.36	57.37 ± 10.70	58.52 ± 10.87	59.86 ± 10.73	
Race (%)					< 0.0001
White	62.65	69.06	71.39	75.55	
Black	14.79	12.78	11.17	9.41	
Mexican American	8.77	7.62	7.07	4.88	
Other Hispanic	8.31	5.79	5.59	4.85	
Other ethnicity	4.75	4.78	5.31	4.75	
Education (%)					< 0.0001
Lower than high school	19.32	17.44	18.14	19.65	
High school	23.03	23.77	24.34	29.35	
More than high school	57.65	58.79	57.52	51.00	
Physical activity (%)					0.0003
Sedentary	16.76	18.83	19.53	21.00	
Low	30.41	29.66	27.86	32.35	
Moderate	20.86	20.25	18.98	20.15	
High	31.97	31.26	33.63	26.50	
Body mass index (kg/m^2^)	27.50 ± 7.65	28.20 ± 7.32	28.55 ± 7.08	28.96 ± 6.43	< 0.0001
HDL cholesterol (mg/dl)	51.77 ± 12.55	56.26 ± 15.08	59.10 ± 16.24	58.74 ± 17.61	< 0.0001
Triglycerides (mg/dl)	89.37 ± 48.36	114.99 ± 63.80	126.22 ± 66.92	179.69 ± 156.35	< 0.0001
LDL cholesterol (mg/dl)	78.47 ± 15.99	104.54 ± 15.45	124.25 ± 16.46	160.34 ± 28.07	< 0.0001
Serum phosphorus (mg/dl)	3.72 ± 0.53	3.73 ± 0.53	3.76 ± 0.52	3.76 ± 0.56	0.0320
Serum calcium (mg/dl)	9.36 ± 0.37	9.41 ± 0.37	9.46 ± 0.37	9.54 ± 0.38	< 0.0001
Serum potassium (mg/dl)	3.93 ± 0.32	3.98 ± 0.34	4.00 ± 0.33	4.02 ± 0.34	< 0.0001
Alkaline phosphatase (U/L)	65.17 ± 23.61	66.97 ± 22.52	69.07 ± 25.43	73.96 ± 28.46	< 0.0001
Protein intake (mg/d)	70.65 ± 31.32	69.74 ± 30.40	69.58 ± 29.25	67.08 ± 29.83	0.0044
Phosphorus intake (mg/d)	1155.78 ± 504.56	1131.99 ± 491.24	1139.46 ± 469.89	1094.96 ± 477.19	0.0027
Lumbar spine BMD (g/cm^2^)	1.07 ± 0.14	1.06 ± 0.14	1.04 ± 0.15	1.01 ± 0.15	< 0.0001

Mean ± SD for continuous variables and *P* value was calculated by weighted linear regression model. % for Categorical variables and *P* value was calculated by weighted chi-square test.

### Univariate analysis

The results of univariate analysis were shown in Table 2. The results of univariate analysis showed that age, race, triglycerides, HDL cholesterol, serum phosphorus, serum calcium, serum potassium, alkaline phosphatase, and LDL cholesterol were negatively correlated with higher lumbar spine BMD. We also found that education levels, physical activity, protein intake, phosphorus intake, and BMI were correlated with higher lumbar spine BMD.

**Table 2:** The result of univariate analysis.

	**Statistics**	**Effect size (β)**	***P* value**
Age (years)	48.51 ± 17.89	– 0.0027 (– 0.0029, – 0.0025)	< 0.0001
Race			
White	3327 (46.91%)	Reference	
Black	1520 (21.43%)	0.0835 (0.0729, 0.0940)	< 0.0001
Mexican American	1643 (23.17%)	– 0.0236 (– 0.0369, – 0.0103)	0.0005
Other Hispanic	333 (4.70%)	– 0.0183 (– 0.0326, – 0.0040)	0.0121
Other ethnicity	269 (3.79%)	– 0.0226 (– 0.0381, – 0.0071)	0.0042
Education			
Lower than high school	2139 (30.20%)	Reference	
High school	1674 (23.64%)	0.0336 (0.0232, 0.0440)	< 0.0001
More than high school	3269 (46.16%)	0.0524 (0.0433, 0.0616)	< 0.0001
Physical activity			
Sedentary	1794 (26.6093%)	Reference	
Low	1960 (29.0715%)	0.0241 (0.0138, 0.0343)	< 0.0001
Moderate	1205 (17.8730%)	0.0347 (0.0235, 0.0460)	< 0.0001
High	1783 (26.4462%)	0.0349 (0.0247, 0.0451)	< 0.0001
Body mass index (kg/m2)	28.93 ± 7.08	0.0037 (0.0032, 0.0041)	< 0.0001
HDL cholesterol (mg/dl)	56.57 ± 15.95	– 0.0007 (– 0.0009, – 0.0004)	< 0.0001
Triglycerides (mg/dl)	134.28 ± 99.63	– 0.0002 (– 0.0002, – 0.0001)	< 0.0001
Serum phosphorus (mg/dl)	3.74 ± 0.54	– 0.0102 (– 0.0166, – 0.0037)	0.0020
Serum calcium (mg/dl)	9.44 ± 0.39	– 0.0432 (– 0.0523, – 0.0342)	< 0.0001
Serum potassium (mg/dl)	3.98 ± 0.34	– 0.0251 (– 0.0354, – 0.0149)	< 0.0001
Alkaline phosphatase (U/L)	73.14 ± 28.54	– 0.0009 (– 0.0011, – 0.0008)	< 0.0001
Protein intake (mg/d)	67.27 ± 30.58	0.0004 (0.0003, 0.0005)	< 0.0001
LDL cholesterol (mg/dl)	118.73 ± 35.83	– 0.0006 (– 0.0007, – 0.0005)	< 0.0001
Phosphorus intake (mg/d)	1086.98 ± 485.35	0.0001 (0.0001, 0.0002)	< 0.0001

### Association between serum TC and lumbar spine BMD

Three weighted multivariate linear regression models were constructed (Table 3). In the unadjusted model, serum TC was negatively correlated with lumbar spine BMD [β = −0.0006, 95% CI: (– 0.0007, – 0.0005)]. After adjusting for confounding factors, this negative association remained in Model 2 [β = – 0.0003, 95% CI: (– 0.0004, – 0.0002)] and Model 3 [β= – 0.0167, 95% CI: (– 0.0334, – 0.0001)]. After converting serum TC from a continuous variable to a categorical variable (quartiles), individuals in the highest quartile had a 0.0135 g/cm^2^ lower lumbar spine BMD than those in the lowest quartile of serum TC.

**Table 3:** Association of serum TC with lumbar spine bone mineral density in 7,092 female participants aged 20–85 years.

	**Model 1**	**Model 2**	**Model 3**
	**β (95% CI) *P* value**	**β (95% CI) *P* value**	**β (95% CI) *P* value**
Total cholesterol (mg/dL)	– 0.0006 (– 0.0007, – 0.0005)***	– 0.0003 (– 0.0004, – 0.0002)***	– 0.0167 (– 0.0334, – 0.0001)*
Q1	Reference	Reference	Reference
Q2	– 0.0172 (– 0.0270, – 0.0073)***	– 0.0049 (– 0.0143, 0.0045)	– 0.0034 (– 0.0229, 0.0160)
Q3	– 0.0343 (– 0.0442, – 0.0244)***	– 0.0066 (– 0.0162, 0.0031)	– 0.0043 (– 0.0304, 0.0218)
Q4	– 0.0691 (– 0.0789, – 0.0593)***	– 0.0301 (– 0.0399, – 0.0202)***	– 0.0135 (– 0.0524, 0.0253)
*P* for trend	< 0.0001	< 0.0001	0.5943

Model 1: no covariates were adjusted. Model 2: age, and race were adjusted. Model 3: age, race, education, physical activity, triglycerides, HDL cholesterol, serum phosphorus, serum calcium, serum potassium, alkaline phosphatase, LDL cholesterol, phosphorus intake, or protein intake were adjusted. The stratified variable was not adjusted again in the model.

*P < 0.05, **P < 0.01, ***P < 0.001. *P* < 0.05 was considered statistically significant.

### Subgroup analyses

On subgroup analysis (Table 4), we observed the association between serum TC and lumbar spine BMD stratified by demographic variables. When stratified by age, a significant negative association existed in participants aged ≧45 years [– 0.0286 (– 0.0528, – 0.0045)]. BMI was converted into categorical variable using 24.9 and 29.9 kg/m^2^ as cut points. When stratified by BMI, serum TC was negatively correlated with lumbar spine BMD in participants with BMI < 24.9 kg/m^2^ [– 0.0280 (– 0.0568, – 0.0008)]. Interaction analyses revealed the association between serum TC levels and lumbar spine BMD was modified by age and BMI (Table 4). The association between TC and lumbar spine BMD was stronger among participants aged over 45 years (β = – 0.0286, *P*_int_ = 0.0049), among participants with BMI < 24.9 kg/m^2^ (β = – 0.0280, *P*_int_ = 0.0055).

**Table 4:** Subgroup analysis of serum TC with lumbar spine bone mineral density, stratified by age and body mass index.

**Subgroup analysis**	**β (95% CI) *P* value**	***P* for interaction**
Age, years		0.0049
Age < 45	– 0.0108 (– 0.0345, 0.0128)	
Age ≧ 45	– 0.0286 (– 0.0528, – 0.0045)*	
BMI (kg/m^2^)		0.0055
< 24.9	– 0.0280 (– 0.0568, – 0.0008)*	
24.9–29.9	0.0059 (– 0.0250, 0.0368)	
>29.9	– 0.0114 (– 0.0391, 0.0163)	

Each stratification adjusted for all the factors (age, race, education, physical activity, triglycerides, HDL cholesterol, serum phosphorus, serum calcium, serum potassium, alkaline phosphatase, LDL cholesterol, phosphorus intake, or protein intake) except the stratification factor itself.

^*^P < 0.05, ^**^P < 0.01, ^***^P < 0.001. *P* < 0.05 was considered statistically significant.

## Discussion

In the present cross-sectional study, we used the representative samples of the NHANES 1999–2006 to analyze the association between serum TC and lumbar spine BMD in US adult women. We found that serum TC were negatively correlated with lumbar spine BMD, especially in women aged over 45 years with BMI < 24.9 kg/m^2^.

Osteoporosis is a metabolic disease, resulting in a progressive reduction in bone strength and enhanced bone fragility with susceptibility to fractures (18). Decreased BMD is an important diagnostic criterion for osteoporosis. The World Health Organization (WHO) defines osteoporosis as a BMD that lies 2.5 SDs below the mean maximum BMD (19). In recent years, there is growing epidemiological and biological evidence supports correlation between osteoporosis and cardiovascular disease (20, 21). Lipid metabolism is involved in the progress of these two disease (20). Among lipid profiles, serum TC is a derivative of cyclopentane dihydrophenanthrene, which is an important participant in the metabolism of tissue cells in the body. Studies show that cholesterol and its metabolites inhibit osteoblastic differentiation in bone cells (22). Moreover, a recent meta-analysis including 33 studies reported that statins increase BMD at the total hip and lumbar spine and decreased the risk of fractures (23). Collectively, these observations seem to suggest that serum TC might serve as a possible biomarker for predicting osteoporosis.

Clinical investigations of the association between serum TC and BMD in adults are controversial. In a cross-sectional study conducted in the USA, researchers discovered a negative correlation between serum TC level and left arm BMD, left leg BMD, and total BMD (10). A Camargo cohort study from Spanish found that serum TC was positively correlated to BMD at lumbar spine and hip (24). Brownbill et al. reported a positive correlation between high level of TC and BMD in 136 Caucasian postmenopausal women (13). Other studies from US also obtained different results regarding the association between serum TC and BMD (14, 25). The population in this study was a representative and large sample of US women aged 20–85 years. Our findings were similar with previous studies (10, 26, 27). The results indicate that serum TC was negatively correlated with lumbar spine BMD in US adult women.

Obesity has been demonstrated to be associated with abnormal lipid metabolism (28). BMI is the most widely used standard for measuring general obesity (29). Some studies reported that BMI was independently related to BMD (30, 31). Another cross-sectional study reported a negative association between waist circumference and lumbar BMD in premenopausal and postmenopausal women with BMI < 25 kg/m^2^, and middle aged men with BMI ≥30 kg/m^2^ (32). Similarly, we converted BMI into a categorical variable using 24.9 and 29.9 kg/m^2^ as cut points. When stratified by BMI, serum TC was negatively correlated with lumbar spine BMD in participants with BMI < 24.9 kg/m^2^. Further prospective intervention studies are need to confirm this association.

Age was an important factor in this study that we could not ignore. Osteoporosis is much more common in women than in men. Approximately, one in three women and one in five men over the age of 50 have osteoporosis (33). In women, rapid bone loss occurs in the early postmenopausal period and lasts for 5–10 years after menopause, suggesting that estrogen loss is an important etiologic factor for osteoporosis in women (34). Several studies have shown a significant increase in serum TC in early menopause (35). The findings of these researches indicated that age is closely related to BMD and lipid metabolism. In subgroup analysis, we found that a higher serum TC level was associated with lower lumbar spine BMD in women aged over 45 years. Therefore, these present results further illustrated the association between lipid metabolism and BMD.

The strength of this study is that the NHANES database contains representative samples of the multi-ethnic population. In addition, the large sample size allows us to better conduct subgroup analyses. However, several limitations needed to be acknowledged in our study. First, the nature of the cross-sectional design makes it difficult to determine the causal relationship between serum TC and lumbar spine BMD. Therefore, further prospective studies are needed to confirm the relationship between serum TC and lumbar spine BMD. Second, the lumbar spine, the hip and the femoral neck are the most commonly used regions for evaluation of BMD. However, BMD of the hip and the femoral neck were not available in most participants and we only examined the association between serum TC and lumbar spine BMD. Finally, there may be other incomplete or unmeasured confounding variables that could alter the results we observed.

In summary, this cross-sectional study discovered a negative association between serum TC and lumbar spine BMD, especially in women aged over 45 years with BMI < 24.9 kg/m^2^. It is suggested that the measurement of serum TC may provide information for predicting poor bone health outcomes in these women.

## Data availability statement

Publicly available datasets were analyzed in this study. This data can be found here: www.cdc.gov/nchs/nhanes.

## Ethics statement

The studies involving human participants were reviewed and approved by board of the National Center for Health Statistics. All the participants provided their written informed consent to participate in this study.

## Author contributions

WF and PP wrote the article. FX collected the data. WH, QW, and MH designed the study. All authors contributed toward data analysis, drafting and critically revising the paper, and agreed to be accountable for all aspects of the work. All authors contributed to the article and approved the submitted version.

## Funding

This study was supported by grants from the project of the National Natural Science Foundation of China (grant numbers 82274544, 81873327, 82004392, and 81573996), the Double First-class Discipline Construction Project of Guangzhou University of Chinese Medicine (grant number Z2015002), the Major Project of Double First-class and High-level University Discipline Collaborative Innovation Team of Guangzhou University of Chinese Medicine (grant number 2021XK05), the Cultivated Project of Double First-class and High-level University Discipline Collaborative Innovation Team of Guangzhou University of Chinese Medicine (grant numbers 2021XK41 and 2021XK46), and the Foundation of Guangdong Educational Committee for Youth Scientists (grant number 2019KQNCX017).

## Conflict of interest

The authors declare that the research was conducted in the absence of any commercial or financial relationships that could be construed as a potential conflict of interest.

## Publisher's note

All claims expressed in this article are solely those of the authors and do not necessarily represent those of their affiliated organizations, or those of the publisher, the editors and the reviewers. Any product that may be evaluated in this article, or claim that may be made by its manufacturer, is not guaranteed or endorsed by the publisher.
